# Visual analysis of blow molding machine multivariate time series data

**DOI:** 10.1007/s12650-022-00857-4

**Published:** 2022-07-11

**Authors:** Maath Musleh, Angelos Chatzimparmpas, Ilir Jusufi

**Affiliations:** 1 Institute of Visual Computing and Human-Centered Technology, TU Wien, 1040 Vienna, Austria; 2 Department of Computer Science and Media Technology, Linnaeus University, Växjö, 351 95 Sweden

**Keywords:** Time series data, Unsupervised machine learning, Visualization

## Abstract

**Abstract:**

The recent development in the data analytics field provides a boost in production for modern industries. Small-sized factories intend to take full advantage of the data collected by sensors used in their machinery. The ultimate goal is to minimize cost and maximize quality, resulting in an increase in profit. In collaboration with domain experts, we implemented a data visualization tool to enable decision-makers in a plastic factory to improve their production process. The tool is an interactive dashboard with multiple coordinated views supporting the exploration from both local and global perspectives. In summary, we investigate three different aspects: methods for preprocessing multivariate time series data, clustering approaches for the already refined data, and visualization techniques that aid domain experts in gaining insights into the different stages of the production process. Here we present our ongoing results grounded in a human-centered development process. We adopt a formative evaluation approach to continuously upgrade our dashboard design that eventually meets partners’ requirements and follows the best practices within the field. We also conducted a case study with a domain expert to validate the potential application of the tool in the real-life context. Finally, we assessed the usability and usefulness of the tool with a two-layer summative evaluation that showed encouraging results.

**Graphical Abstract:**

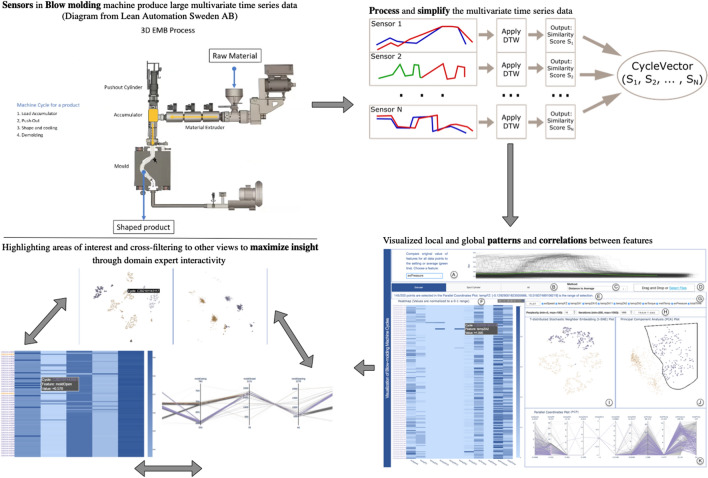

## Introduction

Modern production lines accommodate a high number of sensors and actuators with the aim of improving the quality and reliability of products, enhancing the efficiency of maintenance routines, and ensuring the proper working conditions for several machines (Glebke et al. 2019). These devices generate a wide variety of data ranging from environmental data (e.g., temperature and weather conditions) to data about the production process and the production state (e.g., if a machine is on or off). Analyzing historical data originating from the aforementioned sources can help to accurately forecast whether there will be a change in production speed due to external reasons (Tao et al. 2018).

**Fig. 1:**
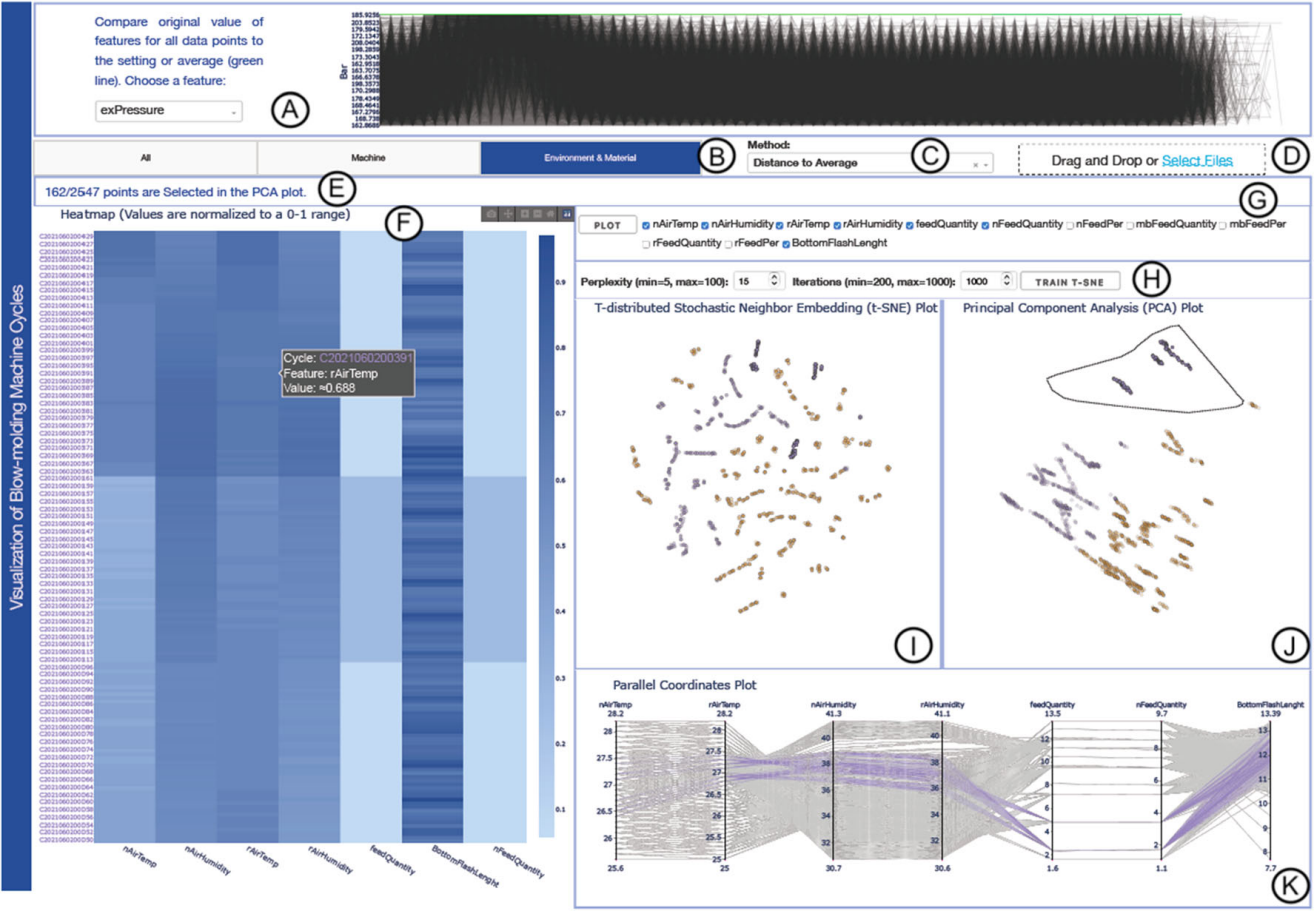
Visualization of machine cycles with the multiple coordinated views of our tool. **(A)** The line plot presents the original values of different features in time series of the production cycle. **B**–**D** panel for the exploration of distinct data subsets with alternative methods. **(E)** The message banner to track the user’s selections. **(F)** The Heatmap displays the normalized values of the DTW-processed time series features. **(G)** The feature selection panel for choosing specific features to plot. **(H)** The user-adjustable t-SNE’s hyperparameters. **I**–**J** The t-SNE and PCA plots form groups of points that can be examined further. **(K)** The PCP highlights the correlation between features. The colors used in **I**–**K** views are computed by applying k-means clustering to the PCA plot

Smart factories rely on careful data management and the choice of appropriate tools for the analysis of data (Park et al. 2016). However, collecting and examining the data using statistical or computational approaches do not satisfy the requirements of an agile production environment (Gao et al. 2020). Domain expertise is crucial for data (and model) interpretation (Chatzimparmpas et al. 2020a) as each production line (or even machine) produces different sensor readings depending on many variables-for example, the air pressure at specific parts of a product and the type of the product. Therefore using visual analytics (VA) to involve the domain experts in the data analysis phase is inevitable (Chatzimparmpas et al. 2020b).

The plastic industry employs several data-driven technologies to optimize the production process (Esposito 2019). Smart factories today produce plastic parts more efficiently and cost-effectively than ever before. Blow molding is one of the most common methods in the manufacturing of plastic (Altarazi et al. 2019). For simplicity, this process could be summed up in two stages: melting the parison (i.e., plastic material) and shaping it in the mold by an air blow (Yu and Juang 2010).

Our industry partner develops robotic and machine learning (ML) solutions for such smart factories. One of their clients produces automobiles plastic parts using the blow molding process. Lately, they introduced a product that collects data remotely through the sensors installed in a blow molding machine. The accumulated data provide a potential for valuable insights related to the production process that can be used to assist factory’s management in decision-making. However, the exploration of multivariate time series data captured by the sensors poses a challenge (Zhou et al. 2018). Indeed, temporal data requires thoughtful methods to extract the important features from tightly coupled multidimensional data. Finally, settling on the optimal visualization techniques requires an extensive investigation of users’ prior experience and their needs (Bernard et al. 2019).

Based on continuous discussions with our industry partner, we collected the following requirements (**R1–R3**):

- (**R1**) highlight emerging patterns of the production process;
- (**R2**) enable the identification of the important features that heavily affect the production process; and
- (**R3**) cover any remaining gaps of their other deployed ML tools to facilitate even further data-driven decision-making.

To satisfy the previously defined requirements, we present visualization techniques for interactive data analysis of remotely collected data in a plastic production factory. We preprocess the data and allow users to explore particular features using clustering and dimensionality reduction (DR) methods (Sacha et al. 2017), as shown in Fig. 1. Our proposed VA tool enables the factory’s management and technicians to make informed maintenance and production decisions. We follow a user-centric approach by involving a visualization expert and an industry partner from the early stages of development to improve our tool’s design iteratively. Overall, our contributions consist of the following:

1. A data visualization tool that comprises several visual representations arranged in a single webpage in order to sufficiently explore multivariate time series data from a blow molding machine.
2. A hypothetical usage scenario and a case study we performed together with a domain expert to validate the tool’s applicability.
3. An extensive evaluation process followed to assess the usability and usefulness of the tool that presented promising results.

This paper is organized as follows. We first survey the literature for essential work in the field that drives our inquiry in Sect. 2. Then, in Sect. 3, we describe the methods of collecting and processing the data. We also outline the data structures used through the development process. In Sect. 4, we explain the functionalities of the proposed dashboard. Afterwards in Sect. 5, we describe a hypothetical usage scenario and a case study that were designed in collaboration with our industry partners. Section 6 describes the evaluation process and its results. The subsequent Sect. 7 explains the design choices and limitations of our approach. Finally, we conclude this paper in Sect. 8.

## Related work

The dynamic time warping (DTW) method is one of the most popular algorithms used to simplify the representation of time series data. Many variations of this algorithm were developed (e.g., local DTW from Yeh et al. 2019) since Berndt and Clifford (1994) suggested the use of DTW to identify patterns in time series data. The underlying process systematically compares data points between two vectors to find the distance between two time series data sets.

Martins and Kerren (2018) propose the SlideDTW algorithm as a more accurate and less demanding alternative compared to FastDTW (Salvador and Chan 2007), PrunedDTW (Silva et al. 2018), and DTW supported by experiments performed with five data sets. The modified algorithm tackles the DTW problem of being computationally demanding. The outcome is that SlideDTW may be optimal for work on large time series, but DTW remains the best option for smaller time series. Steed et al. (2017) successfully employs DTW for the visual analysis of multivariate time series data in additive manufacturing.

Angelopoulos et al. (2020) reviewed clustering and DR methods for fault detection in the industrial sector. In their survey, they found that PCA (Abdi and Williams 2010) was a popular unsupervised algorithm to monitor the production processes. Moreover, k-means (Jain 2010) was discovered to be faster compared to the hierarchical agglomerative and Gaussian mixture. It was one of the sensitive algorithms in multivariable value fluctuations. Similarly, Gittler et al. (2020) affirmed that k-means is an efficient clustering tool despite its drawbacks. The method is rigid as the number of clusters has to be predetermined, however, coupling t-SNE (van der Maaten and Hinton 2008) with PCA algorithms could overcome its weaknesses.

Chen et al. (2020) implement a cross-filtering (Weaver 2008) interactivity in their dashboard. The authors segment the view and use color-encoding to represent multivariate temporal data. They claim that t-SNE will distort the points at the global dimension. Nonetheless, we deem a better solution is to display multiple perspectives of the data with the help of visualization, clustering, and DR.

Time series visualization represents many challenges, while multivariate time series data add to this complexity (Aigner et al. 2011). Several different approaches have been developed addressing the issues presented in this paper (McLachlan et al. 2008; Stitz et al. 2016; Steed et al. 2017). Fujiwara and Shilpika (2021) present a VA framework for gaining insights from multivariate time series data. The authors aim to address problems due to the complexity of time series data, similar to our case. They use PCA and UMAP (McInnes et al. 2018), which are linear and nonlinear DR methods, respectively. Their approach might distract the user as the features are being represented in different plots. Thus, a parallel coordinates plot (PCP) could be proven a more useful tool for comparison. Furthermore, processing the data with two layers of DR might lead to consecutive losses of information. Consequently, the use of separate DR methods might be beneficial.

Although the line plot seems like the favored technique in detecting anomalies in time series data (Hu et al. 2019; Dornhöfer et al. 2020; Talagala et al. 2020), this visual representation might not be sufficient for an exploratory task. Therefore, several solutions couple a line plot or a scatter plot with a stacking technique (Varela et al. 2019; Park and Jung 2020). However, a drawback is that this approach might be perceptually daunting for inexperienced users of VA tools. Interactive tools with multiple views provide better readability and higher chances of detecting patterns (Nguyen et al. 2020; Li et al. 2019; Pham et al. 2019; Stopar et al. 2019; Dang et al. 2020; Schlegel et al. 2020). Furthermore, users are able to explore the data in an agile manner when the data are visualized from both global and local perspectives (Bernard et al. 2019; Chen et al. 2020; Guo et al. 2020). Following these guidelines of recent works, we have created a unique dashboard for the visual analysis of blow molding machine data.

## Data collection and preprocessing

As mentioned before, the blow molding machine is equipped with sensors to collect readings from the various stages of the production process remotely. We received real data samples *(Data Set 1)* to use in the development and evaluation phases of our tool. It contains information from two main sections. The first section consists of 10 features. In detail, *seven temperature readings* along with *speed, pressure, and torque data* collected from the extruder sensors. For the eject cylinder in the second section, we choose 11 features out of the 14 recorded after a discussion with the domain expert.

After the final evaluation (cf. Sect. 6.2), the prototype was tested on different data structures to verify the re-usability of the VA tool. Minor edits were implemented on the data preparation (for more details, see the next paragraph). However, the workflow and dashboard analytical methods were maintained.

The second data structure (*Data Set 2*) provided by our industry partner evolves from the data structure used in the development and evaluation phase. However, substantial changes were implemented in how the data is registered. Additionally, new features were recorded from newly activated sensors. This data set includes information divided into five main sections: 33 features recording machine-related data, four features recording environment readings, nine production material-related readings, three features on mold state, and four other metadata. The metadata includes the *BottomFlashLength* that records the length of the produced profile using image sensors. This feature will be useful in verifying the tool’s findings later on, especially when combined with the insights gained through the use of the dashboard.

Many of the sensors in the outlined sections above are not activated yet. For the testing phase, we used 23 features from the *machine-related readings*, seven features from the *material-related readings*, and four features from the *environment-related readings*. However, after consultations with the industry partners, we reduced the number of features by calculating the *environmentIndex* and *materialIndex*, which are two representative features used during the analysis of the data. Overall, users can choose to explore the data by focusing on: (1) *Environment & Material*, (2) *Machine*, or (3) *All*.

**Fig. 2:**
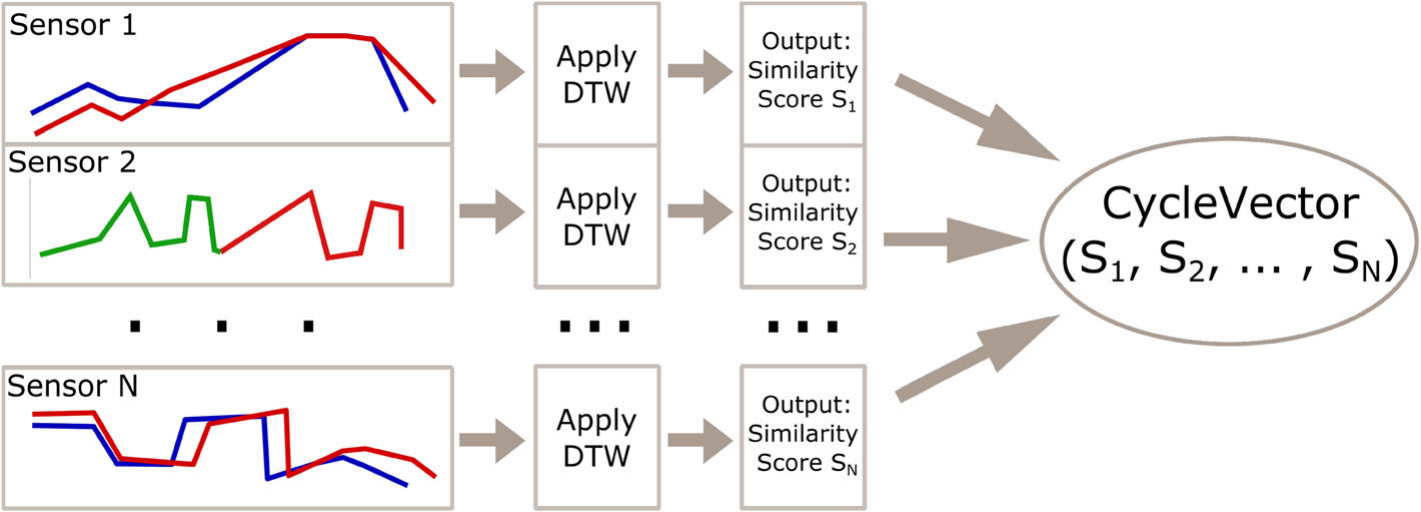
Hypothetical timelines of *N* sensor readings

The term *cycle* in the data describes the loading of a new product material into the accumulator. Each data file represents the information on one cycle (one data point). The cycles are linear and slightly overlapping. Moreover, each cycle is illustrated as multivariate time series data. An important point to notice here is that *ideal* data points are expected for each sensor. This ideal setting is calculated in two ways depending on the sensor in question. The first way is by running some experimental cycles of production where the ideal setting is derived. The second way is by deriving this value based on preceding cycles during production. Normally, the *actual* production readings (in red) differ from the ideal ones (blue lines in Fig. 2). The extent of such difference might influence product quality or even lead to production failure.

Consequently, it is essential to gain insightful information about the differences between the ideal and actual sensor data points, leading us to the next step.

1 $$\begin{aligned}&S_n = f_{DTW}(C_n, I) \end{aligned}$$

2 $$\begin{aligned}&S_n = f_{DTW}(C_n, C_{n-1}) \end{aligned}$$

3 $$\begin{aligned}&S_n = f_{DTW}(C_n, \frac{(C_{n-3} + C_{n-2} +C_{n-1})}{3}) \end{aligned}$$

4 $$\begin{aligned}&S_n = f_{DTW}(C_n, C_{AV}) \end{aligned}$$

Each cycle’s time series data are preprocessed and transformed by the DTW into similarity score (*S*) using one of the four different methods, as seen in (Eqs. 1–4). Thus, a *CycleVector* of sensor similarities is created for each cycle (as seen in Fig. 2). Equation (1) calculates the distance between each cycle’s feature vector (*C*) to the ideal setting value (*I*). In contrast, Eq. (2) calculates distance to the previous cycle (i.e., green line). Equations (3) and (4) calculate the distance to a simple average of the previous three cycles or all cycles, respectively. We also process the data set in terms of product unit derived from its given cycles (i.e., overlaps over two cycles) rather than a single machine cycle.

Our industry partners provided an additional data set (*Data Set 3*) that is structurally very different from the data set we used to develop the tool. The process type of this data set was different than the previous two. This data set recorded observations from the injection molding sensors. The aim was to test the usefulness of the VA techniques in gaining insight in the industrial context regardless of the data structure. The case study present in Sect. 5.2 suggests that our VA tool assists users (in our case, the domain expert) in gaining insights into the data despite the aforementioned process and structural shifts.

This data set is very large, and it recorded the time in millisecond for the opening and closing of the mold separately. Consequently, we had to preprocess the data to ensemble the distinct observations in one coherent data set. It also recorded the time in millisecond that the mold was closed or open during the cycle. The data preparation code was slightly amended to adapt to Data Set 3. The computation time to process this data set was substantially long. It took approximately 57 minutes to process 10,000 data points out of the 4,500,000 available. To develop and test the tool, we used a MacBook Air running on *macOS Big Sur*. This laptop has a *1.6 GHz Dual-Core Intel Core i5* processor, an *Intel HD Graphics 6000 1536 MB* graphics card, and *4GB of 1600MHz DDR3* RAM. Nevertheless, optimizing for data processing time is beyond the testing objectives. Thus, the final data set containing thousands of observations contained five features, and it was analyzed using the same visualization techniques.

## Visual analytics tool

Our methodology includes the end-user in the development process, as explained in Sect. 1. Monthly video meetings scaffold the development of the initial prototype. For this tool, we used Dash Plotly (2015) as a framework and Plotly (2010) for the visualizations. The tool was hosted on a server to facilitate the evaluation phase (cf. Sect. 6).

Figure 1A plots each feature for all cycles plus the *setting* vector in green for comparison. It gives a view of the original data which enables users to take a closer look to deviations within a single feature.

Figure 1B enables users to select the group of features to analyze. In Fig. 1C, users can select one out of the six different methods to simplify the representation of the time series data. Also, a new data set can be uploaded, as shown in Fig. 1D.

The users’ activity is emphasized in a banner that displays textual descriptions of the filters applied at that time (see Fig. 1E). The proposed tool provides feature selection possibilities for knowledgeable users. They can unselect/select the features they wish to explore further (see Fig. 1G). Afterward, the tool reprocesses the data and reclusters the points automatically.

Additionally, users have the option to train t-SNE models based on the adjustments of *perplexity* and *iterations* parameters (cf. Fig. 1H). The default values are 15 for the former and 1,000 for the latter. This feature gives control to the users to try out alternative embeddings. However, it requires as a prerequisite to have experience with t-SNE and its hyperparameter tuning (Chatzimparmpas et al. 2020).

The data are visualized with t-SNE (see Fig. 1I) and PCA algorithms (Fig. 1J). The t-SNE’s and PCA’s features are trained from the normalized values of the selected features. These views can illustrate a general topology of the data set that users can use as a starting point for exploring the data. PCA is a linear method that provides a seemingly straightforward clustering where the first principal components capture most of the data variance. However in highly-exploratory data analysis scenarios, these clusters might not represent what the user is looking for. In contrast, t-SNE is a popular nonlinear method that complements PCA with a different clustering structure focusing on local structures of a data set. Although t-SNE projections might be challenging to interpret and contain many pitfalls (Wattenberg et al. 2016), it is a method that expands the exploratory potential for the user.

We run *k-means* on the PCA features to color-encode the clusters for the DR views. Using the *elbow method* (Nainggolan et al. 2019), we conclude that two clusters (represented in orange and purple colors (Brewer and Harrower 2021)) are optimal in our case. Finally, by clicking on top of these representations, all plots are reset.

*PCP* visualizes the processed features of each cycle, as indicated in Fig. 1K. This view supports the analysis by highlighting the correlation between features.

The x-axes represent the features selected in the analysis, and the y-axes are the range of the processed data for each feature before normalization. Users can select different ranges and rearrange the position of the features in the PCP. In addition, a *Heatmap* allows the comparison of the active features for the normalized values (cf. Fig. 1F). The x-axes exhibit the selected features for analysis, while the y-axes depict the ordered cycles. The color scale in blue reflects the range from 0 to 1.

Cross-filtering functionalities are available for most of the plots. Users can select points in any of the two projections using a lasso (e.g., Fig. 1J, in black). This interaction leads to filtering of the selected points on the other embedding, the PCP, and the Heatmap views. Brushing and linking also work through PCP selections.

## Applications

In this section, we describe a hypothetical usage scenario and a case study that showcase the potential application of our tool. The data used for those two applications is real-life data from the partner company. The usage scenario (i.e., Sect. 5.1) explores feature-related insights from *Data Set 2*, and the case study (cf. Sect. 5.2) identifies the causes of failure using *Data Set 3*. Both use cases were drafted in collaboration with our partners at Lean Automation. The first out of the two was designed based on discussions in the early phases of the development. It was later updated according to the new data sets received from Lean Automation.

In detail, we conducted a walk-through session with John Mikkelä, the chief technical director at Lean Automation. He has many years of experience as an automation engineer and maintenance manager. During the implementation of the tool, he was the lead technician working with the development of analysis and optimization tools for a blow molding machine factory.

### Usage scenario: exploring the impact of features

In a hypothetical scenario, Maria is a technician with many years of experience working for a plastic factory. She wants to examine all the data recorded by the blow molding machine between June 1–3. She recognizes that the quality of the products varies, and she needs to understand which features have the highest impact on the production quality. She drops the data file containing the improved Data Set 2 into the upload box in Fig. 1D.

Initially, she is interested in exploring deviations and impacts of the detailed environment and material data on the production quality. Thus, she selects the Environment and Material tab, as illustrated in Fig. 1B. She further would like to calculate the clusters based on the distance to the average in data sets (Fig. 1C). As seen in Fig. 1G, she only plots the seven features of interest based on her previous experience.

She selects three nearby groups of points in Fig. 1J to observe in Fig. 1K any interesting anomalies that may occur. As seen in Fig. 1J, she chooses the points that seemed to belong to a dispersed cluster in the upper right corner using the lasso functionality. She hopes that this interaction can tell her something about the optimal value ranges for the features and highlight any possible deviations. She notices that the t-SNE plot (Fig. 1I) reveals a few distinct clusters in the upper bound. Therefore, she expects some variance in the feature values (**R1**).

With the initial PCA selection of 162 points, the technician examines the PCP (see Fig. 1K). She knows from experience that changes in the *Air Humidity* might affect the quality of the parison. She reorders the vertical axes (features) to better understand relationship between features. From the *BottomFlashLength* axis, she selects all instances with deviation values up to $\approx$13.39. Although the *BottomFlashLength* values are distributed, they tend to average higher than normal. From experience, she assumes that the humidity indicator should be high when the air temperature indicator is low, and vice-versa. In this particular cluster, the readings indicate a possible issue. This corresponds to a lower observation in the *FeedQuantity*. She makes a selection on the PCP and drags it to observe how the relationship between features changes (**R2**).

Afterward, the technician confirms her findings by examining the Heatmap in Fig. 1F. For the selected points, the values of *nAirTemp*, *rAirTemp*, and *BottomFlashLength* fluctuate between cycles. However a pattern emerges, she recognizes that *feedQuantity* and *nFeedQuantity* increase when *nAirTemp* values are lower. Maria further examines the *exPressure* feature in the line plot (cf. Fig. 1A). She notices deviations in a certain block with higher values (visible in the left-hand side of the line plot). Furthermore, she zooms into the line plot to note the period upon which the deviation took place. Accordingly, the technician plans ahead for a closer inspection of units produced in the deviating cycle mentioned above, and she orders a further investigation of the cause behind the variations in the readings of the *the Temperature and Humidity sensors*. She uses the insight to adjust models in the other installed ML and artificial intelligence (AI) tools (**R3**).

Maria concludes that there must be more parameters that influence the production processes. The interesting question for her is which feature has a higher impact on the production result. An indicator of the feature impact on the *BottomFlashLength* will direct her to better spot the area of the problem. Thus, it will help her stabilize the production process, and subsequently the production quality.

### Case study: diagnosing the causes of failure

**Fig. 3:**
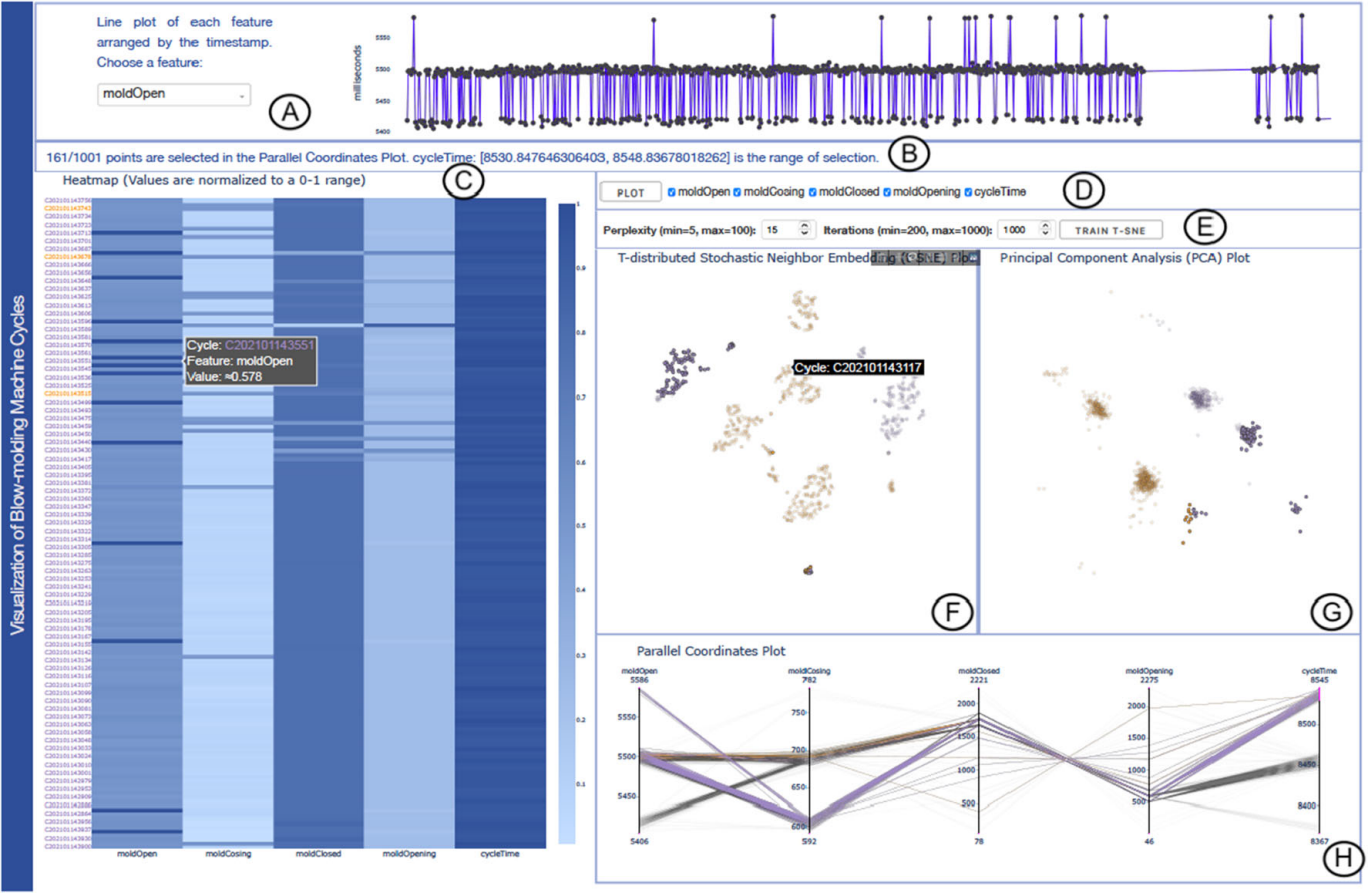
Visualization of Data Set 3 with the multiple coordinated views of our tool. **A** The line plot presents the original values of different features. **B** The message banner to track the user’s selections. **C** The Heatmap displays the normalized values of the DTW-processed time series features. **D** The feature selection panel for choosing specific features to plot. **E** The user-adjustable t-SNE’s hyperparameters. **F**–**G** The t-SNE and PCA plots form groups of points that can be examined further. **(H)** The PCP highlights the correlation between features. The colors used in **F**–**H** views are computed by applying k-means clustering to the PCA plot

John is a quality control manager. He monitors the qualities of profiles produced by the blow molding machine. John is interested in detecting deviations in mold closing and opening intervals during the injection process. A few anomalies could indicate quality issues with the corresponding products. However, a series of consecutive data aberrations would indicate a need for machine maintenance. John would also like to give a rough indication of how the profiles might be clustered before conducting quality tests on samples.

The quality control manager starts by looking at the PCP in Fig. 3H. The cycle time could be an indicator of anomalies. He selects the upper limit of the cycle time. He wants to check where in the cycle the deviation happened. He notices a drop in the mold closing time to 600ms (**R2**). Next, he zooms into the Heatmap in Fig. 3C to detect interesting patterns and confirm relations between the different variables. He also takes a note of the days when the anomalies appeared, as it appears in the cycle name in the label box seen in the Heatmap. He notices that something has occurred on a specific day (**R1**).

After exploring the relation between the different variables on the PCP and confirming patterns in the Heatmap, he decides to dig deeper into the data. He believes that an issue rose during the mold closing process that needs to be investigated. Furthermore, he wants to get a quick overview to see at what time did the deviation pattern occur. John surveys the line plot in Fig. 3A to detect the period of the deviations. He checks each observation to detect the anomalies. He notices that the major deviations occurred between 8:45 am and 9:45 am (**R3**).

John successfully detected at what stage of the process the issue happened. He also observed that when the mold closing time is longer the mold closed time tend to be shorter, and vice versa (**R2**). He concludes that the injection time is probably faster when the mold closing time is longer. The material in the cylinder stays a longer time, and thus, it flows easier when it is injected (**R1**). This indicates that there might be some quality issues. The mold closing time and mold closed time should be consistent from cycle to cycle to reach consistency in the production quality. The process at the moment is not stable. He decided to order the installation of more sensors to observe the mold closing process and detect the exact nature of the problem. He also moved to further analyze the quality of the products produced during the time period where deviations occurred. It will give him a better picture of the impact of these deviation on the production quality (**R3**).

## Evaluation

There are many different approaches and guidelines for evaluating VA tools/systems and visualization approaches (Carpendale 2008; Sedlmair et al. 2012; Sedlmair 2016; Rogers et al. 2021). Since our tool evolves as our industry partner extends the number of sensors and subsystems installed in the production lines, we evaluate it using a formative usability evaluation method (Maner 1997; Lazar et al. 2017). This method guarantees a constant flow of feedback throughout the development process. After that, we conducted postdevelopment summative evaluation sessions to assess the usability and usefulness of the visualization tool.

### Formative evaluation

In detail, we conducted three evaluation sessions in five months. In each session, the VA tool was assessed by a visualization expert (**E1**) and an industry partner (**E2**). The former is a coauthor of this paper, however, he was unaware of this fact up until the first two evaluations. The experts used the tool on their personal machines via an online link, and they had to follow a set of concrete instructions divided into three core stages.

In the *first stage*, they tested the solution based on a checklist of all possible functionalities to identify logical errors. The *second stage* is related to benchmarking and satisfaction of users’ expectations in general. In the *third stage*, the focus is on the experts’ impressions about the tool.

In the first evaluation session, although no bugs were found, **E1** highlighted numerous logical issues in cross-filtering processes. He also found various inconsistencies in the colors of the plots. We selected a diverging color scheme that is colorblind safe to avoid deceptive perception issues (Brewer and Harrower 2021). Thus, the user interface (UI) was updated to resolve those problems. **E2** reported that “the ability to easily filter and select which data to analyze” is the most important functionality of this tool. Moreover, **E1 and E2** wanted to have the ability to associate the processed values to the real-world data. We added this functionality in the last version of the tool.

**Both experts** reported a satisfactory improvement of the dashboard in every way and excellent time responsiveness for the second version of the tool. However, **E1** said that the Heatmap seemed “disconnected from the other visualizations.” Thus, we used orange and purple colors for the y-axis text labels to correspond to the other plots’ clusters (cf. Fig. 1). Additionally, **E2** emphasized the need to “test the tool in real-life situations” to assess it better. Finally, he recommended implementing the ability to upload new data sets.

As for the last session, **E2** was impressed with the updates. Particularly, the new functionality of choosing different methods for comparing the original values of the various features visually is a game-changer because it fits the analytical workflow of our tool. **E1** pointed out to few logical flaws with the functionalities of the dashboard, namely the dropdown menu. Specifically, he said that: “it did not synergize well”. Furthermore, several aesthetic suggestions were provided with an utter goal to improve the perception of the tool. We plan to consider them in a future design iteration of our dashboard. Although, a start was made with the implementation of many formatting and styling fixes for the final prototype.

The GUI’s compactness was improved to fit all views within a typical computer’s monitor margins. Furthermore, **E2** gave us more feedback on how to increase the usefulness of the tool. He recommended a customized UI for mobile screens as he sees a value-added in controlling the process through mobile devices. Furthermore, he expressed the importance of adding a data live-streaming functionality to the dashboard. At the moment, the prototype analyzes historical data only. Both suggestions are deemed as potential paths for future improvements of the VA tool.

### Summative evaluation

Finally, we conducted a summative evaluation with five experts. We requested qualitative feedback from three additional VA researchers and two domain experts (one of them was **E2**). The five participants filled out the ICE-T evaluation form (Wall et al. 2019), and they answered the system usability scale (SUS) questionnaire (Brooke 1996). Overall, the results were positive and encouraging, as shown in Table 1.

The ICE-T table contains responses for each component from strongly disagree (in dark purple) to strongly agree (in dark green), with 4 in white color being the neutral answer. For the SUS table, two inverse legends are utilized as questions are alternatively worded positively and negatively in order to minimize bias. After reversing the scores of the negatively worded questions and shifting the scores to a 0 to 4 scale, the scores are added and then multiplied by 2.5 to calculate the percentiles as seen in the Score column (Brooke 1996).

**Table 1:**
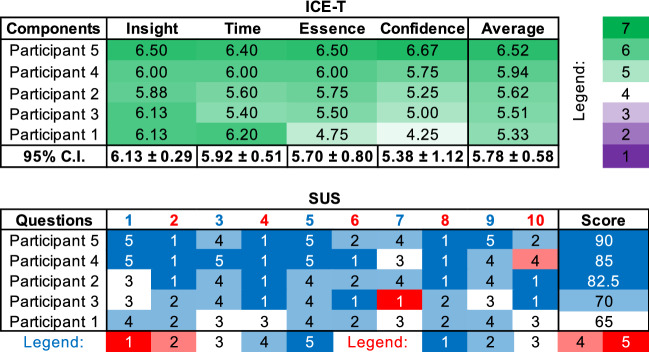
Results from the ICE-T and SUS feedback forms. In the ICE-T table, 7 denotes the most positive response and 1 the least positive. In the SUS table, 5 represents the strongest agreement with the statement, while 1 is the strongest disagreement

The summative evaluation of the tool pointed out that industrial knowledge is crucial in perceiving the usefulness of the tool. This outcome matches our expectations due to the exploratory nature of the problem at hand. Thus, the solution was rated higher by participants with industry experience, especially on the confidence criteria. The results of the ICE-T survey imply that the domain-related learning curve should be improved in future iterations. As suggested by *Participant 1* incorporating tooltips and guidance tools in the dashboard could help in achieving the aim of making the solution more user-friendly. The VA expert confirmed that the proposed solution: “supports exploratory analysis very effectively.” This evaluation was supported by the industry expert who expressed their confidence that the tool: “provides useful interactive capabilities to help investigate the data in multiple ways” and “allows quality decision-making based on data”. *Participants 5* re-affirmed the importance of developing a live-analytical functionality within the dashboard (as was already mentioned in the past). In his final comment, *Participant 4* (who is an industry and VA expert) described our solution as: “straight to the point, very user friendly, and effective.”

## Discussion

In this section, we will recap the main lessons learned, discuss our design choices, and present the limitations of the tool. We designed the dashboard to provide an easy-to-learn tool without compromising the depth of analysis it delivers. In the process, we encountered several limitations that impacted our decisions.

### Lessons learned

Our implementation experience with this tool outlines the importance of developing flexible VA solutions that could adapt to different data structures. The continuous changes in the structure of the data set received from our partner presented challenges regarding the confirmation of our tool’s usefulness. Hence, involving our partner/domain expert in the development process was deemed extremely valuable. This collaboration did not only allow us to design a tool that satisfies the partners’ requirements, but it also played a crucial role in shaping and articulating those requirements in the first place.

The formative evaluation was a great facilitator for the communication with domain experts. It allowed us to get a better understanding of the domain problems, and it was a great opportunity to introduce and explain VA approaches to domain experts and get a quick feedback from them. Finally, another important aspect is the several layers of evaluation described in Sects. 5 and 6 which could be considered as a qualitative rating of our tool’s effectiveness.

**Summary** The two applications indicate that the developed dashboard provides potential for use in the industry. The suggested workflow of the analysis that takes the user on an analytical journey between a global and local analysis of the data has the potential to maximize the data insight in an exploratory context of industrial data. The results signify that the developed solution model can provide insights efficiently. The VA workflow could be adapted to several analytical contexts in the plastic blow molding industry and beyond. We tested the usefulness and reusability of our VA tool by implementing it on two different data sets as outlined in Sect. 3. By maintaining the overall VA approach, we wanted to verify that our tool enables users to gain insights into an industrial process with minimal changes to the dashboard and code. We received the two data sets from our industry partners after the end of the evaluation. One data set is an upgraded version of the data set we used in the development phase. The other data set had a completely new structure. We conducted minimal changes to the code to adapt to the new data structures. We later met on a video link with our industry partners to verify the usefulness of the dashboard. Our partners communicated their confidence that the tool provides valuable insights into the production process.

### Design choices

The dashboard provides a compact view that facilitates users to stroll between the different views. The scatter plots, providing a global view of the data, were placed on top of the PCP that provides a local perspective. This choice is meant to adapt to a top-down exploration model that is more suitable for a user’s task of exploring the data with little information on what to look for.

We believe that adding a guiding component to the scatter views would enhance the usability of the tool. Users with no background of the t-SNE and PCA dimensionality reduction methods might find these views confusing to use. In the PCP, we modified the plot to maintain grayed-out lines of the unselected data points. This feature provided the users with the useful functionality of comparing the path of the selected against the unselected data points. Thus, the detection of the reasons behind identified anomalies becomes more efficient, as evident with the case study in Sect. 5.2. Although one inherent problem with the PCP is the limited number of features that can be visualized concurrently without introducing cluttering/overlapping issues, we have managed to partially address this issue with the support of filtering features (e.g., see Fig. 1G).

### Limitations

Unfortunately, one limitation of the case study conducted in Sect. 5.2 was the available computation resources due to the processing power of the laptop used while experimenting with the dashboard. Since Data Set 3 contains millions of rows of records, we tested our tool on a sample of the entire data set. Despite that, we used sequential observations rather than random to maintain the patterns. Deploying the visualization in a cloud server will alleviate this problem, especially if combined with progressive VA workflows (Stolper et al. 2014). We plan to incorporate such solutions and further explore several fragments of this data set in the near future.

Another limitation is that to better tune the tool, we have to set up our dashboard to work in the real production environment. This plan was interrupted by the spread of COVID-19, limiting us to distance evaluation and testing on historical data. Furthermore, the current tool is sensitive to the data structure. In the absence of data collection standards, the tool would require an extra effort to adapt the dashboard to the context. Nevertheless, the code structure minimized this effort.

By the time we conducted the summative evaluation, the tool was not optimized for the variability of screen resolutions. Thus, this could have contributed to the variance in user experience during the sessions described in Sect. 6.2. Furthermore, the pandemic restrictions limited our ability to visit a plastic factory to recreate a real-life scenario. Therefore, the use cases were prepared after an extensive discussion we had with our partners at Lean Automation rather than with a technician in the plastic factory.

Finally, although the limited number of experts available to evaluate the tool constraints the generalization of our approach (see Sect. 6.2), the ICE-T methodology suggests that five participants is the bare minimum for obtaining valid results (Wall et al. 2019). Furthermore, the standard deviations in all components of the ICE-T table appear relatively low, which can be considered as indicative of reaching a certain consensus (cf. Table 1, 95% C.I.). The most fluctuating value is $\pm 1.12$ which refers to the participants’ confidence in the results produced from the use of our dashboard. For the SUS table, three participants gave similar (and extremely positive) scores, but the last two gave neutral or slightly positive feedback (see Table 1, Score column). By taking into account these findings, we conclude that the evaluation sessions would possibly lead to more valuable feedback if more industry experts were involved at that stage. One of our future goals is to validate further our work with additional experts in the real production environment.

## Conclusions

In this paper, we employed several VA techniques to provide a plastic factory’s technicians with valuable insights from a blow molding machine sensory data. We developed an interconnected dashboard that uses the DTW method to preprocess the data, t-SNE and PCA approaches to reduce the dimensionality of our data, and k-means for clustering all available cycles into two clusters. Finally, the evaluation sessions yielded encouraging results.

In the future, the firm plans to install several cameras throughout the production process that will use image recognition to measure the quality of the products for classifying them. Connecting the new data to the patterns emerging from our tool is a future direction of research.
